# Monitoring of Selected Health Indicators in Children Living in a Copper Mine Development Area in Northwestern Zambia

**DOI:** 10.3390/ijerph14030315

**Published:** 2017-03-19

**Authors:** Astrid M. Knoblauch, Mark J. Divall, Milka Owuor, Colleen Archer, Kennedy Nduna, Harrison Ng’uni, Gertrude Musunka, Anna Pascall, Jürg Utzinger, Mirko S. Winkler

**Affiliations:** 1 Swiss Tropical and Public Health Institute, P.O. Box, CH-4002 Basel, Switzerland; juerg.utzinger@unibas.ch (J.U.); mirko.winkler@unibas.ch (M.S.W.); 2 University of Basel, P.O. Box, CH-4003 Basel, Switzerland; 3 SHAPE Consulting Ltd., GY1 2 St Peter Port, P.O. Box 602, Channel Islands; mdivall@shapeconsulting.org (M.J.D.); mowuor@shapeconsulting.org (M.O.); 4 University of Kwa Zulu Natal, Durban 4041, South Africa; archerc@ukzn.ac.za; 5 Solwezi District Health Management Team, Solwezi 40100, Zambia; kennedy.nduna@yahoo.com (K.N.); h.nguni@gmail.com (H.N.); 6 First Quantum Minerals Limited, Lusaka 10100, Zambia; gertrude.musunka@fqml.com (G.M.); anna.pascall@fqml.com (A.P.)

**Keywords:** anaemia, health impact assessment, hookworm, malaria, migration, stunting, Zambia

## Abstract

The epidemiology of malaria, anaemia and malnutrition in children is potentially altered in mining development areas. In a copper extraction project in northwestern Zambia, a health impact assessment (HIA) was commissioned to predict, manage and monitor health impacts. Two cross-sectional surveys were conducted: at baseline prior to project development (2011) and at four years into development (2015). Prevalence of *Plasmodium falciparum*, anaemia and stunting were assessed in under-five-year-old children, while hookworm infection was assessed in children aged 9–14 years in communities impacted and comparison communities not impacted by the project. *P*. *falciparum* prevalence was significantly higher in 2015 compared to 2011 in both impacted and comparison communities (odds ratio (OR) = 2.51 and OR = 6.97, respectively). Stunting was significantly lower in 2015 in impacted communities only (OR = 0.63). Anaemia was slightly lower in 2015 compared to baseline in both impacted and comparison communities. Resettlement due to the project and migration background (i.e., moving into the area within the past five years) were generally associated with better health outcomes in 2015. We conclude that repeated cross-sectional surveys to monitor health in communities impacted by projects should become an integral part of HIA to deepen the understanding of changing patterns of health and support implementation of setting-specific public health measures.

## 1. Introduction

Solwezi district in the Northwestern Province of Zambia has traditionally been a rural, sparsely populated area [1]. However, recent mining developments (i.e., the Kansanshi and Lumwana copper mines) have accelerated in-migration and altered the socioeconomic profile of the district [2,3]. In 2009, the Trident project—a copper mine operated by First Quantum Minerals Limited (FQML)—was launched [4,5]. The mine, which became operational in 2015, is a green field development in a previously remote forested area, covering a lease area of approximately 950 km^2^. The development included construction of an open pit mine, processing plant, power lines, airstrip, maintenance and administrative infrastructure, access roads and a new residential settlement for the mine workforce and their families. The project development spurred socio-demographic and economic changes in the local community, including physical resettlement, influx of job- and opportunity-seeking migrants, shift in livelihood strategies and urbanization [6,7]. Hence, the direct and indirect ecological, social, economic and health impacts placed on the communities living in this area have been considerable [8,9].

Traditionally, studies determining community health impacts associated with mining have focused on HIV and other sexually transmitted infections (STIs), tuberculosis, water and air quality or exposure to hazardous chemical substances [10,11]. Furthermore, malaria is often considered by companies operating in the tropics because of its significant contribution to the local burden of disease and workplace health implications [12]. However, it is less evident how conditions that are especially prevalent in children living in low- and middle-income countries, such as anaemia, diarrhoeal diseases, respiratory tract infections, intestinal parasitic infections or malnutrition are affected by project-related transformations over longer periods of time.

Health impact assessment (HIA) is the recommended approach to predict potential effects of industrial projects on the health of affected populations by considering a broad range of social, cultural, economic and ecological determinants of health [13,14]. As part of the Trident project’s feasibility studies, an HIA was commissioned to assist in the identification of potential health impacts and development of a community health management plan to prevent adverse health impacts and maximize health benefits. During the scoping phase of the HIA, a number of health data gaps were identified, which warranted additional primary data collection [15,16]. Hence, a cross-sectional baseline health survey (BHS) was conducted in 2011 [17]. Data from the BHS and secondary data sources (e.g., local health statistics) provided an evidence-base for the subsequent risk assessment phase of the HIA [18]. Therefore, the identified potential health impacts were ranked based on their significance (i.e., impact severity and likelihood of occurrence) using a semi-quantitative risk-ranking matrix [19]. A community health management and monitoring plan was developed that combines continuous and periodic data collection approaches, including district health information system data and repeated cross-sectional health surveys at four-year intervals. While some diseases warrant continuous surveillance depending on their aetiology and significance (e.g., HIV), repeated cross-sectional household surveys at 3–5-year intervals measuring key health indicators are a valid option to observe conditions in communities that may change over longer periods of time (e.g., stunting) and also to allow for assessment of true prevalences as well as knowledge, attitudes and practices (KAP) [20,21,22].

Here, we present data from two cross-sectional epidemiological surveys: the 2011 BHS, prior to project development, and the first follow-up health survey completed in 2015, hence, four years into project development. Among the broad spectrum of indicators assessed, Table 1 summarizes the ones selected based on their significance for child health and relevance in the current project setting. The paper specifically discusses trends over the four-year period and makes comparisons between impacted communities (i.e., affected by the project development) and non-impacted comparison communities, and describes associated determinants at household and community levels.

## 2. Materials and Methods

### 2.1. Ethical Considerations

The study protocols for the two cross-sectional surveys received approval from the ethics review committee of the Tropical Disease Research Centre (Ndola, Zambia; registration number 00003729). The Solwezi District Health Department supported the studies as a key government partner, with contributions to the study design, community sensitization and fieldwork. At the household level, informed consent (signed or fingerprinted) was obtained from heads of households or mothers/caregivers. At the school level, sensitization activities included school visits prior to the survey. Teachers were informed about the objectives and procedures of the study and consent was obtained by teachers informing parents about the study, who in turn provided written permission to allow their children to participate in the survey. Children assented orally. Children who were found positive for *Plasmodium falciparum* infection using a rapid diagnostic test (RDT) were treated with an artemisinin-based combination therapy, using artemether-lumefantrine, following national protocols. Children found with mild and moderate anaemia (haemoglobin (Hb) 7–11.0 g/dL) were provided with iron and multivitamin supplements, while severe cases (Hb < 7 g/dL, or those with any signs/symptoms of severe anaemia) were referred to the nearest health facility thereby adhering to the public health referral system followed in Zambia. All children who provided stool specimens for parasitological testing were given a single oral dose of albendazole (400 mg).

### 2.2. Study Area and Community Profile

**Figure 1:**
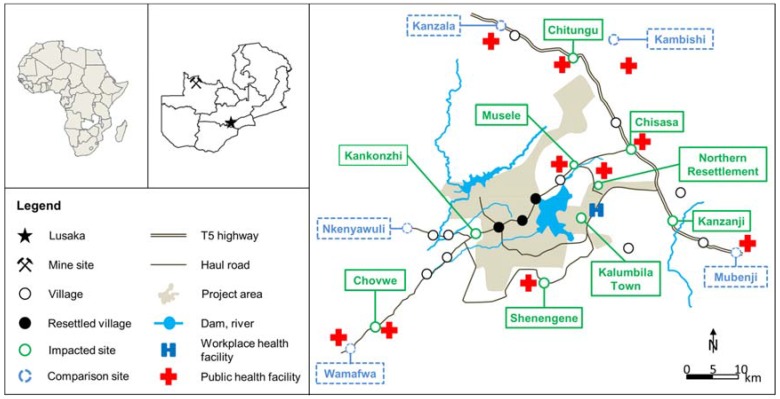
Study area and sentinel sites, Trident project, 2011 and 2015, Zambia.

The Trident project is located about 150 km northwest of Solwezi town, the district capital (Figure 1). Chisasa is the major settlement in the study area, at the junction along the T5 highway connecting Solwezi to Mwinilunga district. At the time of the BHS in 2011, over 60% of the adult population was involved in subsistence agriculture and about 2% employed by the project [6]. In 2015, about 35% of the households in the impacted sites had at least one member employed by, or working as a subcontractor for, the project [7].

### 2.3. Study Design and Sampling Method

Two cross-sectional, epidemiological surveys were conducted in July 2011 and July 2015, using the same methodology. Considering the heterogeneity in the distribution of project-related health impacts expected across communities, a stepwise, semi-purposive sentinel site sampling strategy, rather than a fully randomised design, was employed in both surveys [17]. In a first step, all villages potentially affected by the project were identified, whereas “potentially” refers to an impact that may or may not occur and “affected” refers to being affected by either a direct impact (e.g., resettlement or project-sponsored health interventions) or an indirect impact (e.g., in-migration along transport corridors) caused by the project [8]. In a second step, impacted sites were semi-purposively selected based on the magnitude and nature of project-related impacts (e.g., resettled communities and communities along transport corridors). Our approach allowed for sampling of smaller, potentially impacted communities that might otherwise have been excluded, had a random cluster sampling proportional to population size been employed [35]. In a third step, comparison sites were chosen based on their socio-demographic and topographic similarity to the impacted sites as well as proximity to the project area, with two inclusion criteria: (i) located outside the project area; and (ii) no or only limited project-associated impacts such as no project-sponsored health interventions in the community or project employees/contractors residing in the community. In the final step, households were randomly selected within the sentinel sites, with the inclusion criteria at the level of the household requiring the presence of a mother (≥15 years) with at least one child under the age of five years. In parallel, schoolchildren from primary schools in the sentinel sites were sampled to screen for hookworm infection, the most prevalent soil-transmitted helminth in the study area [36].

**Figure 2:**
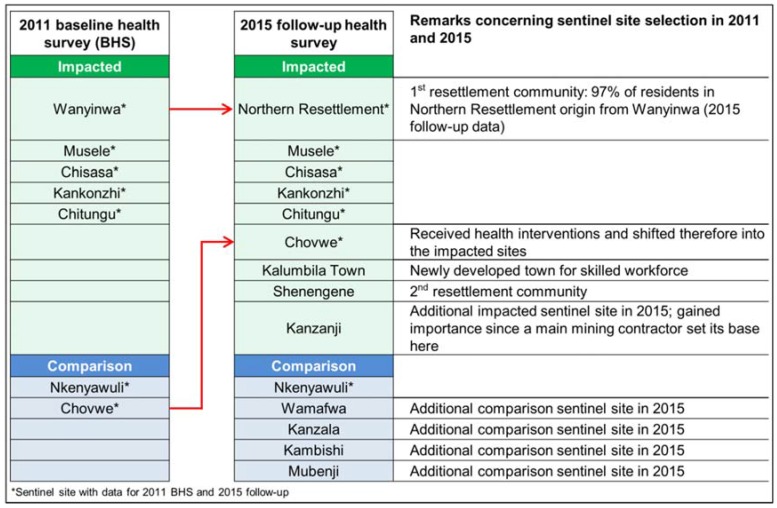
Sentinel site selection, Trident project, 2011 and 2015, Zambia.

The full list of sentinel sites selected for the 2011 BHS and the 2015 follow-up is shown in Figure 2. In seven sentinel sites, data were collected in both the BHS and follow-up. For an additional seven sentinel sites, data were only available for 2015. This included two impacted sites: the newly developed Kalumbila Town (employee residential area) and Shenengene (a resettlement village). One impacted site was added due to increased importance (Kanzanji became the base of a major mining contractor) and four additional comparison sites were included to augment statistical power for comparison in future surveys. Importantly, findings from Wanyinwa (sampled during the 2011 BHS) are comparable to findings from Northern Resettlement (sampled during the 2015 follow-up) as 97% of the participating households in Northern Resettlement originated from Wanyinwa.

### 2.4. Data Collection

The surveys included two main data collection methods: (i) a questionnaire interview with caregivers (≥15 years) in the household; and (ii) an assessment of biomedical indicators in children under the age of five years in a mobile field laboratory. The questionnaire focused on KAP related to issues such as health seeking behaviour, maternal and child health, infectious diseases and participation in health interventions. In addition, basic socio-demographic information was collected, including information on recent in-migration (defined as duration of residency in the current location of less than five years). The questionnaire is provided as a supplementary file S1.

On completion of the questionnaire, caregivers together with their under-five-year-old children were asked to visit the field laboratory for the assessment of biomedical indicators. An RDT was used to assess *P*. *falciparum* infection from a finger-prick capillary blood sample in children aged 6–59 months (see Table 1). Hb concentration was measured in a capillary blood sample from children aged 6–59 months to determine anaemia (defined as Hb < 11 g/dL). Children aged < 5 years had their weight and height measured.

At each school enrolled in the survey, a quota of at least 15 boys and 15 girls was randomly selected. Therefore, all eligible children (i.e., present at the day of the survey; aged 9–14 years) were listed and numbered and the quota was selected using random number sampling. A fresh morning stool sample was collected and subjected to the Kato-Katz technique. A single 41.7 mg thick-smear was examined within 20–40 min for enumeration of hookworm eggs [34]. Eggs were counted and multiplied by a factor of 24 to determine eggs per gram of stool (EPG).

### 2.5. Data Analysis

In 2011, questionnaire data were entered into EpiData software (EpiData Association; Odense, Denmark). In 2015, data were collected through electronic tablets using the open data kit (ODK) software. Analysis was performed with Stata (StataCorp LP, College Station, TX, USA). Frequencies and odds ratios (ORs) with corresponding 95% confidence intervals (CIs) were determined. Mixed effects logistic regression models were used taking into account clustering at the levels of sentinel sites and of households. The model included a factor for year to capture potential period effects, a factor for type of site (impacted vs. comparison) and an interaction term between the two factors to assess potential differences in changes of prevalence rates from 2011 to 2015 between impacted and comparison sentinel sites. Of note, for 2011 and 2015 comparisons, only sentinel sites that were sampled in both surveys were considered. For analysis with 2015 data only, all 14 sentinel sites were considered.

## 3. Results

### 3.1. Study Population

The study populations in 2011 and 2015 are shown in Table 2. In 2011, 289 households were sampled from seven sentinel sites, and in 2015, 516 households were sampled from 14 sentinel sites, with a total sample of 483 and 949 children under the age of five years, respectively. Additionally, 309 (2011) and 477 (2015) children aged 9–14 years were sampled from the selected schools. For 2015 only, the proportions of household with resettlement or migration background and the proportion of households using improved sanitation facilities are shown.

### 3.2. P. falciparum Infection Prevalence

At baseline, children in impacted sites showed a lower odds for *P*. *falciparum* infection (OR = 0.33, 95% CI 0.05–2.20; Table 3). There was a significantly higher prevalence in 2015 compared to 2011 in all sites and overall in both the impacted and comparison sites, with ORs of 2.51 (95% CI 1.56–4.02) and 6.97 (95% CI 2.20–22.0), respectively, but with no significant different period effect between impacted vs. comparison (OR = 0.36, 95% CI 0.10–1.23).

**Figure 3:**
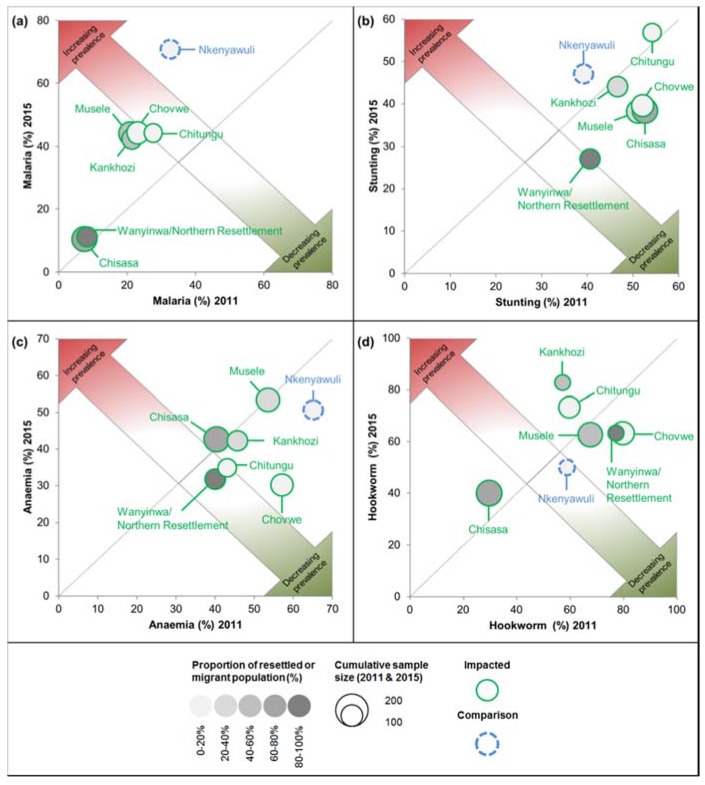
Prevalence rates per sentinel site, Trident project, 2011 and 2015, Zambia: (**a**) prevalence of *P*. *falciparum* in children aged 6–59 months; (**b**) prevalence of stunting in children aged 0-59 months; (**c**) prevalence of anaemia in children aged 6–59 months; and (**d**) prevalence of hookworm in children aged 9–14 years.

**Figure 4:**
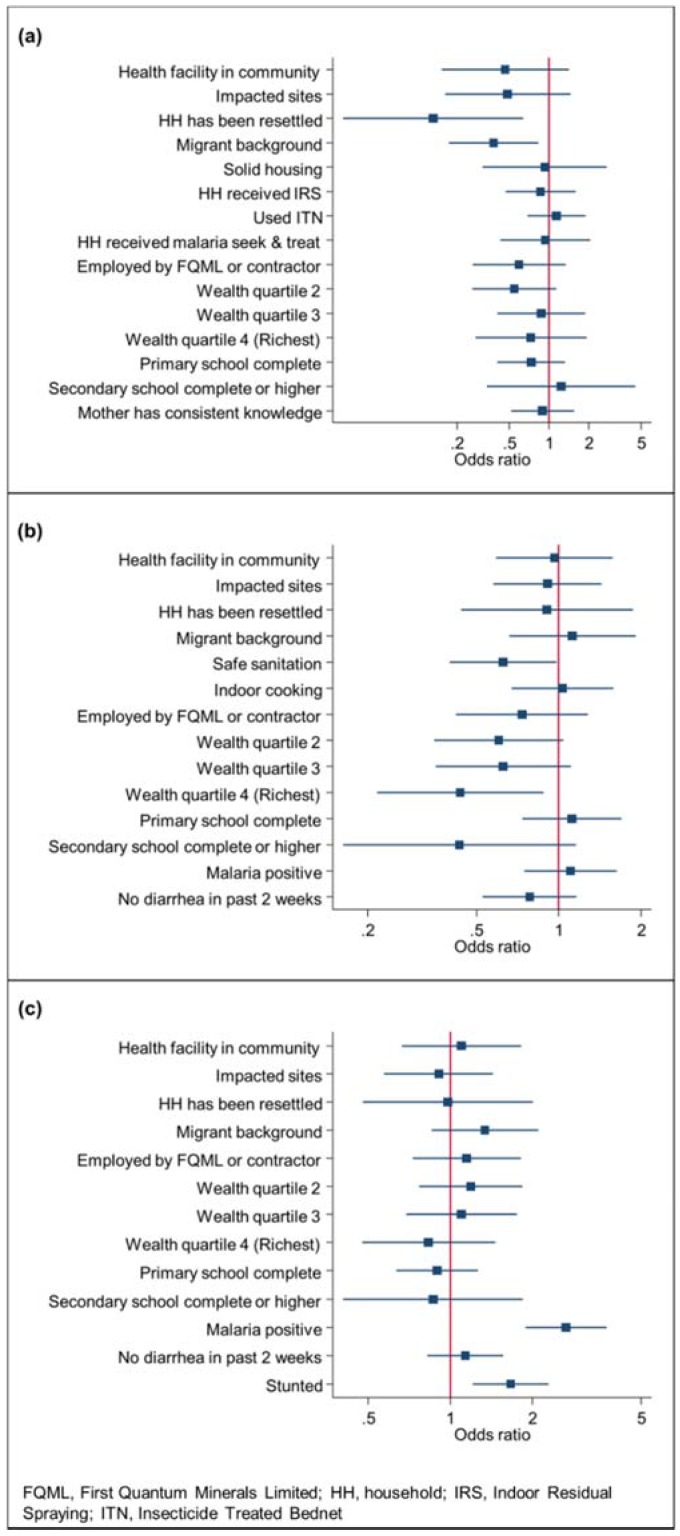
Determinants of health outcomes during the 2015 follow-up health survey, with adjusted odds ratios and 95% confidence intervals, Trident project, Zambia: (**a**) determinants of *P*. *falciparum* in children aged 6–59 months; (**b**) determinants of stunting in children aged 0–59 months; and (**c**) determinants of anaemia in children aged 6–59 months.

In Figure 3a, the prevalences of 2011 (x-axis) and 2015 (y-axis) are plotted against each other. Communities whose prevalence has increased are plotted in the upper left half of the graph coloured in red and communities whose prevalence has decreased are plotted in the lower right half of the graph coloured in green. Communities whose prevalence has remained stable are located on, or close to the grey line. Wanyinwa/Northern Resettlement and Chisasa were least affected by *P*. *falciparum* infection in both 2011 and 2015. In 2015, both communities exhibited high proportions of resettled households and new settlers and, as illustrated in Figure 4a, children with a resettlement or migration background had significantly lower odds of being infected with *P*. *falciparum*.

### 3.3. Stunting Prevalence

At baseline, the stunting rate was slightly higher in the impacted compared to the comparison sites but with no statistical significance (OR = 1.61, 95% CI 0.77–3.35; Table 3). In 2015, stunting was significantly lower in the impacted sites compared to 2011 (OR = 0.63, 95% CI 0.46–0.87), whilst in the comparison sites stunting was higher in 2015 (OR = 1.41, 95% CI 0.58–3.46).

Two factors significantly lowered the risk for stunting in the 2015 study population: (i) access to improved sanitation facilities; and (ii) originating from the richest wealth quartile (Figure 4b). In 2015, children from Northern Resettlement were least affected by stunting (Figure 3b).

### 3.4. Anaemia Prevalence

While the difference in anaemia prevalence between impacted and comparison sites was significant at baseline (*p* = 0.04), there were no significant changes over time in the two categories of sites, although it decreased in both; from 46.6% (194/416) to 41.9% (173/413) in the impacted and from 65.1% (28/43) to 50.8% (33/65) in the comparison sites, respectively (Table 4). Anaemia prevalence was lower in 2015 in all but two sentinel sites (i.e., Chisasa and Musele), where it remained stable (Figure 3c). In 2015, factors significantly associated with anaemia in a child were a concurrent *P*. *falciparum* parasitaemia and stunted growth (Figure 4c).

### 3.5. Hookworm Infection Prevalence

The overall prevalence of hookworm infection slightly decreased from 62.5% (172/275) to 60.9% (145/238) in the impacted sites and from 58.8% (20/34) to 50.0% (15/30) in the comparison sites. Hence, the rates of infection did not change significantly over time (*p* = 0.71 and *p* = 0.47, respectively; Table 4). Chisasa had the lowest infection rate in 2015, which was however higher than the rate recorded in 2011 (Figure 3d).

## 4. Discussion

Presented here is a selection of indicators in children from two cross-sectional surveys spaced by four years within the frame of the Trident copper development project in Zambia. Living in an impacted sentinel site or in a resettled household was associated with better health outcomes for *P*. *falciparum* infection, anaemia and stunting in under-five-year-old children. Improved health outcomes were reported in association with distal factors such as employment or relative household wealth, suggesting that the project development may result in positive effects on the health status of children.

The most noticeable change observed was the higher prevalence of the *P*. *falciparum* infection rate in 2015 compared to 2011 in all sentinel sites. Nkenyawuli, the only comparison site sampled in both 2011 and 2015, showed a markedly higher prevalence in 2015 compared to the impacted sites. Malaria control interventions have been implemented by the project and district health management teams in the impacted sentinel sites, including indoor residual spraying (IRS), distribution of long-lasting insecticidal nets (LLINs), education and awareness and ‘malaria seek and treat’ (i.e., active case detection and treatment performed through house-to-house visits at weekly intervals) [37]. These interventions were generally associated with lower odds for *P*. *falciparum* infection. Children in resettled households showed significantly lower *P*. *falciparum* infection rates in 2015. In the newly built settlements of Northern Resettlement and Shenengene, prevalences were lowest at 10.9% and 6.3% in 2015, respectively, with the new, solid housing structures having closed eaves and window screens that are associated with lower infection risk as shown before in other malaria-endemic settings [38]. When excluding resettled or migrant households, no other factor was found a determinant for *P*. *falciparum* infection (see supplementary Figure S2). Nevertheless, across the entire study area, the 2015 follow-up showed higher *P*. *falciparum* infection prevalence compared to the 2011 baseline. This observation is in line with a wider trend in Northwestern Province found during two consecutive Malaria Indicator Surveys (MIS). Indeed, the prevalence in under-five-year-old children, as assessed by RDT, was 17.3% in 2010, while it was almost double in 2012 (32.5%) [23,39]. The strong increase coupled with the absence of significant associations with common risk factors at household and community level point to an environmental influence. As both surveys were conducted in July, we speculate that there were considerable inter-annual fluctuations, such as changes in the average temperature or precipitation [40,41].

The stunting rate in children is influenced by a multitude of factors such as recurrent infectious diseases (e.g., hookworm infection), persistent enteropathy, access to improved sanitation and safe drinking water, access to food or children migrating from areas with different rates of stunting [42,43]. Overall, the stunting rates in 2015 in the impacted (39.4%) and comparison sites (47.0%) were similar or higher than the average of the Northwestern Province (36.9%), as determined during the 2013/14 Demographic and Health Survey (DHS) [29]. The improvement of stunting between 2011 and 2015 was significant in the impacted sites but not in the comparison sites. Of all the determinants assessed during the 2015 follow-up, wealth and access to improved sanitation were associated with lower stunting rates. Wealth remained a determining factor when excluding resettled or migrant households as well as households with safe sanitation (see supplementary Figure S2). Access to improved sanitation and reduced environmental contamination has been found previously to avert stunting in children [44]. Among the sentinel sites visited in both surveys, Northern Resettlement, where new houses were built with adjoining ventilated improved latrines, had consequently the highest proportion of households with access to safe sanitation in 2015 (97.1%; Table 2) and at the same time the lowest stunting rate.

Anaemia rates in impacted (41.0%) and comparison sites (49.4%) in 2015 were comparable to data obtained during the 2012 MIS for the Northwestern Province, where anaemia was reported in 45.5% in under-five-year-old children [23]. These high rates of anaemia will continue to have long-lasting negative consequences in the study area given that iron deficiency undermines growth, physical fitness and educational performance [30]. Malaria and stunting remained significant determinants for anaemia in multivariate regression models where resettled and migrant households were excluded, respectively (see supplementary Figure S2). This high anaemia rate is a concern, particularly if one considers that health facilities were present in 11 of the 14 sentinel sites (Figure 1) and that health facilities could be most efficient in combating anaemia through the provision of primary health care services, including antimalarial drugs, iron supplementations and growth monitoring [30,45].

To our knowledge, no survey data on soil-transmitted helminths for Solwezi district are publicly available. A recent geostatistical analysis by Karagiannis-Voules et al. (2015) estimated the prevalence of soil-transmitted helminth infections at 50% or higher in the general population in that area, which is in line with our findings (50% in the impacted and 60.9% in the comparison sites, respectively) [36]. Hookworm was the predominant soil-transmitted helminth species in both surveys, with similar prevalences in 2011 and 2015. Most children (94.3%) had mild-to-moderate infection intensities (i.e., <4000 EPG; data not shown) and hookworm infections are therefore expected to play an immaterial role in anaemia burden in the current setting [46]. According to the Solwezi District Health Management team, preventive chemotherapy using albendazole was done seven months prior to each survey—in December 2011 and December 2014. However, breaking transmission of hookworm will remain difficult when children continue to walk barefoot, and hence, are in contact with hookworm egg-contaminated soil in this setting [47].

Migrant populations can be especially vulnerable to ill-health as they face restricted social cohesion and exclusiveness leading to inequalities [48]. However, in the current setting, children with a recent migrant history were generally found in better health than those from host communities. This can be partly explained by the fact that the migrants in this area were labour- or opportunity-seekers as opposed to involuntarily displaced people. For example, migrant children had significantly lower *P*. *falciparum* infection than children who were born and lived in the study area all along. Interestingly, *P*. *falciparum* infection prevalence differed greatly between Kalumbila Town (43.2%) and Chisasa (10.4%), the two settings with the highest proportions of migrant households (100% and 65.8%, respectively). While in Chisasa most migrant children came from within Solwezi district (40.9%) or other places in the Northwestern Province (28.8%), most migrant children in Kalumbila Town stem from the Copperbelt Province (43.2%) or Lusaka (8.1%), which are low prevalence areas [23]. For anaemia, however, rates were higher in 2015 compared to 2011 in Chisasa only and remained stable in Kankhozi and Musele, the three sentinel sites with higher proportions of migrants. Potentially new infectious diseases or sudden changes in lifestyle (e.g., feeding habits) coupled with a limited awareness of, and capacity to address, anaemia within the household could explain the slightly higher rates in the migrant population.

The noted differences between migrant and host population of children illustrate the importance of understanding the characteristics of migrant populations (e.g., origin, level of skills, health status, economic means and reasons for migration) and their interplay with the local communities. Despite this, they remain often neglected in HIA, especially when planning public health interventions [49].

The lack of baseline health data is an inherent limitation for monitoring of health in communities subjected to natural resource development and management projects in low- and middle-income countries [50,51]. For the Trident project, the BHS completed in the frame of the HIA provided a strong evidence-base that reflected the health status of communities prior to project development. Supported by this evidence-base, the HIA identified a wide range of health conditions that warranted management and monitoring throughout the project lifecycle. A priority was given to the control of STIs, including HIV, based on the perceived significant impact, whereas the outcomes of mitigation activities are publicly shared elsewhere [52]. In the absence of a regulation that requires transparent dissemination of HIA outcomes, presenting the findings in the peer-reviewed literature provides an opportunity to adhere to good practice standards such as transparency and the ethical use of evidence, while at the same time producing valuable case studies of HIA practice in the context of natural resource development projects in low- and middle-income countries [53,54]

### Limitations

There were no data in 2011 for several sentinel sites that were only added in the 2015 follow-up, which obviously restricts “before–after” comparison. However, the five comparison sites surveyed in 2015 should represent a sufficiently large comparison group for future follow-up surveys. The non-random sentinel site sampling strategy allowed for inclusion of sites considered too important to miss but the resulting non-randomised sample and the results are therefore relevant to the selected sentinel sites only. Due to lower sensitivity of a single compared to duplicate Kato-Katz thick smears, the true hookworm prevalence is likely to be higher than presented here [55]. Furthermore, household characteristics and behavioural aspects (e.g., toilet use at school and footwear) were not determined in children participating in the school survey.

## 5. Conclusions

Children living in villages considered impacted by a copper mine development in Northwestern Province of Zambia showed generally better health outcomes for *P*. *falciparum* infection, anaemia and stunting than children from comparison sites, whereas project-induced changes such as resettlement and employment had a positive influence. These findings though do not infer causality. Through the application of the HIA, health-targets were integrated in a project development that has primarily economic goals, which is in line with the health-in-all sectors approach embraced by the Sustainable Development Goals (SDGs) agenda [56]. Repeated cross-sectional monitoring of key health indicators and determinants of health in communities impacted by projects help to better understand whether and how human health is impacted, which population sub-groups are most vulnerable and help identify underlying risk factors. In collaboration with staff from the local health system, evidence from periodic and longitudinal monitoring generated in the private sector allow for prioritization and adaption of targeted and locally sensitive interventions whereby the public and private sectors share responsibility and synergize efforts in safeguarding human health.

## Figures and Tables

**Table 1:** Selected indicators in children and their relevance in the Trident copper mining project area, Zambia.

Indicator	Definition and Measurement Methods	Relevance to Children’s Health and the Local Project Context
*Plasmodium falciparum* infection prevalence in children aged 6–59 months	*P*. *falciparum* infection is defined as the detection of the *P. falciparum* histidine-rich protein II antigen in capillary blood using a rapid diagnostic test (RDT; SD BIOLINE Malaria Ag P.f; Standard Diagnostics Inc., Gyeonggi-do, Republic of Korea) [23].	Improved local economy, vector control measures implemented by the project and better infrastructure (e.g., roads, health facilities) can improve access to vector control measures and health care [12,24]. Environmental alteration due to project activities can potentially increase the number of vector breeding sites [25,26]. Camp follower settlements may develop with poor associated environmental health conditions potentially increasing vector breeding sites and human-vector contact if not managed appropriately [27,28].
Stunting prevalence in in children aged 0–59 months	Stunting, or low height-for-age, is defined as -2 standard deviation units from the WHO reference population median and measured using a digital scale and portable stadiometer (Seca 877; Seca GmbH, Hamburg, Germany) [29].	Improved local economy can improve nutritional status. Reduced access to agricultural land for local populations and food price inflations due to increased purchasing power can increase the burden of malnutrition
Anaemia prevalence in children aged 6–59 months	Anaemia is defined as haemoglobin (Hb) < 11 g/dL in capillary blood assessed using a HemoCue® 201+ testing device (HemoCue Hb 201 System; HemoCue AB, Ängelholm, Sweden) [23]. Age was recorded based on the date of birth given in the child’s vaccination card, if available, or based on parents report.	Anaemia is used as a proxy indicator for general health and well-being, because of its multifactorial aetiology (e.g., intake and uptake of dietary iron, parasitic infections and prevalence of sickle cell disease) [30,31,32]. Epidemiology of infectious diseases, access to health care and diets potentially change due to the project development which, in turn, influences rates of anaemia [33].
Hookworm infection prevalence in children aged 9–14 years	Hookworm infection is defined as detection of hookworm eggs in a single thick-smear of a fresh, morning stool sample prepared and examined by the Kato-Katz technique within 20–40 min after slide preparation (using 41.7 mg templates) [34]. Intensity of infection was determined by counting hookworm eggs per slide and multiplied by a factor of 24 to obtain eggs per gram of stool (EPG).	Project-induced in-migration may place pressure on existing sanitation, which poses a risk for the transmission of diarrhoeal diseases and intestinal parasites. Increased income coupled with behaviour change can lead to protection through wearing of footwear. First-time inhabitation of native soil (e.g., new settlements or resettlement), increased use of footwear (due to increased income) and intensive circulation of top soil (due to project-associated activities) can lower exposure to hookworm eggs in the environment.

**Table 2:** Study populations, Trident project, 2011 and 2015, Zambia.

Sentinel Sites	Households		Children Aged < 6 Months		Children Aged 6–59 Months		School-Going Children Aged 9–14 Years		Proportion of Households that Have Been Resettled due to the Project	Proportion of Migrant Households (in the Area for <5 years)	Proportion of Households that Use Improved Sanitation
Year	2011	2015	2011	2015	2011	2015	2011	2015	2015	2015	2015
Wanyinwa (2011)/Northern Resettlement (2015)	35	34	4	7	60	63	35	30	97.1	2.9	97.1
Musele ^1^	30	66	2	18	43	116	40	59	3.0	30.3	34.9
Chisasa ^1^	66	65	3	16	94	96	44	60	1.5	66.2	47.7
Kankonzhi ^1^	36	30	3	7	70	52	35	29	3.3	36.7	50.0
Chitungu ^1^	30	33	1	8	58	43	57	30	0.0	0.0	21.2
Chovwe ^1^	61	32	3	10	91	43	64	30	0.0	6.3	43.8
Kalumbila Town	NA	30	NA	7	NA	36	NA	30	0.0	100.0	100.0
Shenengene	NA	32	NA	4	NA	48	NA	30	96.9	3.1	93.8
Kanzanji	NA	32	NA	8	NA	51	NA	29	3.1	43.8	6.3
Total impacted	258	354	16	85	416	548	275	327	19.5	34.5	52.3
Nkenyawuli ^1^	31	32	8	3	43	65	34	30	0.0	6.3	37.5
Wamafwa	NA	33	NA	6	NA	66	NA	30	0.0	6.1	33.3
Kanzala	NA	32	NA	4	NA	52	NA	30	0.0	15.6	21.9
Kambishi	NA	32	NA	8	NA	51	NA	30	0.0	0.0	3.1
Mubenji	NA	33	NA	6	NA	55	NA	30	0.0	21.2	0.0
Total comparison	31	162	8	27	43	289	34	150	0.0	9.9	19.1

^1^ Sentinel site with data for 2011 BHS and 2015 follow-up; NA: not available.

**Table 3:** Prevalences and period effects for *P*. *falciparum* infection and stunting, Trident project, 2011 and 2015, Zambia.

	*P*. *Falciparum* Infection in Children Aged 6–59 Months				Stunting in Children Aged 0–59 Months			
	n	Prevalence (%; 95% CI)	OR	*p*-Value	n	Prevalence (%; 95% CI)	OR	*p*-Value
**Difference at baseline**								
Comparison (2011)	43	32.5 (19.0–48.5)	1.00		51	39.2 (25.8–53.8)	1.00	
Impacted (2011)	416	17.5 (14.0–21.5)	0.33 (0.05–2.20)	0.25	432	49.7 (44.9–54.5)	1.61 (0.77–3.35)	0.20
**Period effect ^1^**								
Comparison (2011)	43	32.5 (19.0–48.5)	1.00		51	39.2 (25.8–53.8)	1.00	
Comparison (2015)	65	70.7 (58.1–81.3)	6.97 (2.20–22.0)	<0.01	68	47.0 (34.8–59.5)	1.41 (0.58–3.46)	0.44
**Period effect ^1^**								
Impacted (2011)	416	17.5 (14.0–21.5)	1.00		432	49.7 (44.9–54.5)	1.00	
Impacted (2015)	413	30.9 (26.5–35.6)	2.51 (1.56–4.02)	<0.01	479	39.4 (35.0–43.9)	0.63 (0.46–0.87)	<0.01
**Change over time in impacted vs. comparison sites**								
Comparison (2011–2015)	n/a	n/a	1.00		n/a	n/a	1.00	
Impacted (2011–2015)	n/a	n/a	0.36 (0.10–1.23)	0.10	n/a	n/a	0.44 (0.17–1.15)	0.09

^1^ Describes the change in prevalence between 2011 and 2015; CI: confidence interval; n: sample size; n/a: not applicable; OR, odds ratio.

**Table 4:** Prevalences and period effects for anaemia and hookworm, Trident project, 2011 and 2015, Zambia.

	Anaemia in Children Aged 6–59 Months				Hookworm in Children Aged 9–14 Years			
	n	Prevalence (%; 95% CI)	OR	*p*-Value	n	Prevalence (%; 95% CI)	OR	*p*-Value
**Difference at baseline**								
Comparison (2011)	43	65.1 (49.0–78.9)	1.00		34	58.8 (40.6–75.3)	1.00	
Impacted (2011)	416	46.6 (41.7–51.5)	0.47 (0.22–0.98)	0.04	275	62.5 (56.5–68.2)	1.16 (0.33–4.03)	0.80
**Period effect ^1^**								
Comparison (2011)	43	65.1 (49.0–78.9)	1.00		34	58.8 (40.6–75.3)	1.00	
Comparison (2015)	65	50.8 (38.0–63.3)	0.55 (0.24–1.22)	0.14	30	50.0 (31.2–68.7)	0.69 (0.25–1.88)	0.47
**Period effect ^1^**								
Impacted (2011)	416	46.6 (41.7–51.5)	1.00		275	62.5 (56.5–68.2)	1.00	
Impacted (2015)	413	41.9 (37.0–46.8)	0.79 (0.60–1.05)	0.11	238	60.9 (54.4–67.1)	1.07 (0.73–1.56)	0.71
**Change over time in impacted vs. comparison sites**								
Comparison (2011–2015)	n/a	n/a	1.00		n/a	n/a	1.00	
Impacted (2011–2015)	n/a	n/a	1.44 (0.62–3.36)	0.39	n/a	n/a	1.54 (0.53–4.46)	0.42

CI, confidence interval; n: sample size; n/a: not applicable; OR, odds ratio; ^1^ Describes the change in prevalence between 2011 and 2015.

## Supplementary Materials

The following are available online at www.mdpi.com/1660-4601/14/3/315/s1, supplementary file S1: The questionnaire focused on KAP related to issues such as health seeking behaviour, maternal and child health, infectious diseases and participation in health interventions, supplementary file S2: Wealth remained a determining factor when excluding resettled or migrant households as well as households with safe sanitation.
