# Supplementary material for: Monitoring of Selected Health Indicators in Children Living in a Copper Mine Development Area in Northwestern Zambia
Source: Int J Environ Res Public Health. 2017 Mar 19;14(3):315. doi: 10.3390/ijerph14030315 (PMC5369151; doi:10.3390/ijerph14030315)
Supplement: Supplementary file 2 [file ijerph-14-00315-s002.docx]

**Supplementary Materials: Monitoring of Selected Health Indicators in Children Living in a Copper Mine Development Area in Northwestern Zambia**

**Astrid M. Knoblauch, Mark J. Divall, Milka Owuor, Colleen Archer, Kennedy Nduna, Harrison Ng’uni, Gertrude Musunka, Anna Pascall, Jürg Utzinger and Mirko S. Winkler**
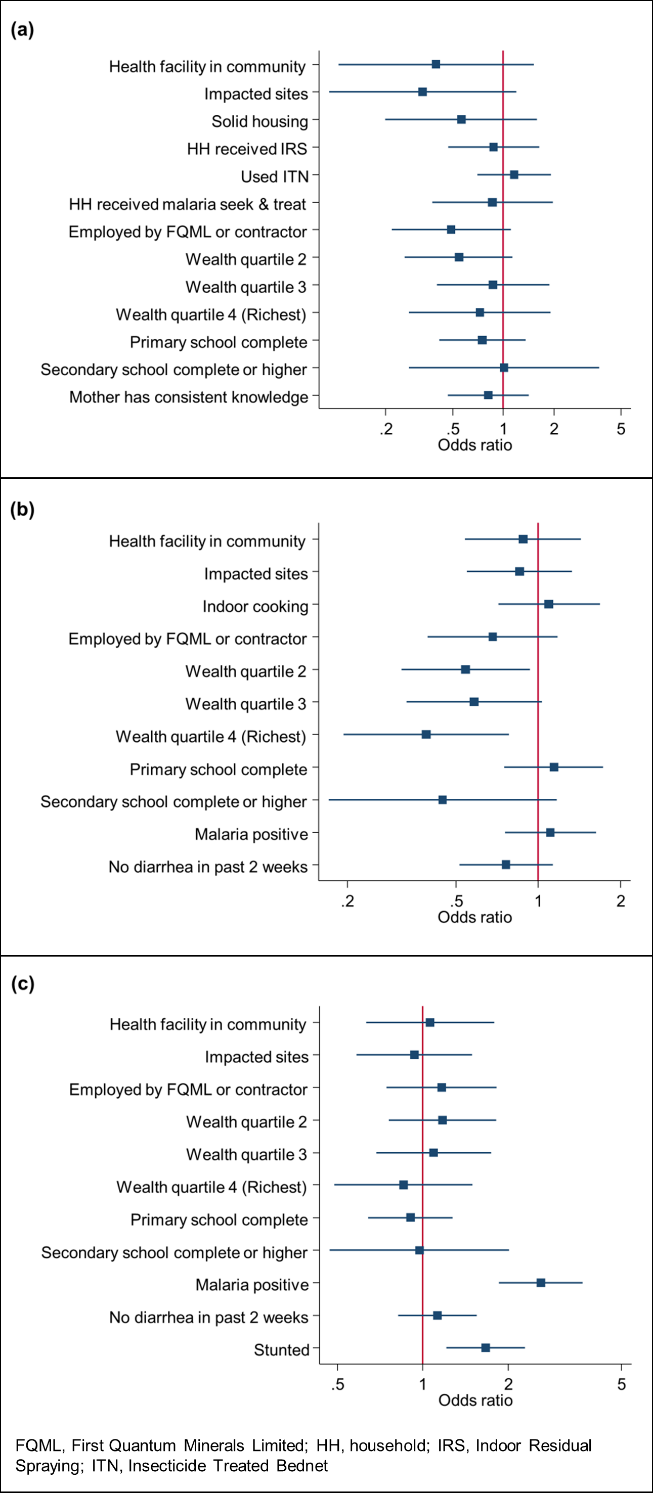


**Figure S2.** Wealth remained a determining factor when excluding resettled or migrant households as well as households with safe sanitation.
